# Over Three Decades of Mental Health in Sub‐Saharan Africa: An Analysis of GBD Data From 1990 to 2023

**DOI:** 10.1155/ghe3/5570226

**Published:** 2026-07-30

**Authors:** Basile Njei, Yazan A. Al-Ajlouni, Samira Y. Lemos, Nkengafac Villyen Motaze, Laurent Cleenewerck de Kiev, Luchuo E. Bain, Yauba Saidu

**Affiliations:** ^1^ Engelhardt School of Global Health and Bioethics, Euclid University, Bangui, Central African Republic; ^2^ Department of Medicine, Section of Digestive Diseases, Yale University, New Haven, Connecticut, USA, yale.edu; ^3^ VA Connecticut Healthcare, West Haven, Connecticut, USA; ^4^ Ohio University Heritage College of Osteopathic Medicine, Athens, Ohio, USA, ohio.edu; ^5^ Yale Liver Center, Yale New Haven Health, New Haven, Connecticut, USA; ^6^ Department of Rehabilitation, Montefiore Medical Center, Bronx, New York, USA, montefiore.org; ^7^ Department of Diabetes and Endocrinology, Yaoundé General Hospital, Yaoundé, Cameroon; ^8^ Medicine Usage in South Africa (MUSA), North-West University (NWU), Potchefstroom, South Africa; ^9^ Euler-Franeker Memorial University (EULER), Willemstad, Curaçao, Netherlands; ^10^ African Population and Health Research Center, APHRC, Nairobi, Kenya; ^11^ Department of Psychology, University of Johannesburg, Auckland Park, South Africa, uj.ac.za; ^12^ Clinton Health Access Initiative, Yaoundé, Cameroon; ^13^ Institute for Global Health, University of Siena, Siena, Italy, unisi.it; ^14^ Department of Environmental & Global Health, University of Florida, Gainesville, Florida, USA, ufl.edu

**Keywords:** disability-adjusted life years, Global Burden of Disease, health policy, mental disorders, Sub-Saharan Africa

## Abstract

**Background:**

Mental disorders are leading causes of disability worldwide but remain underprioritized in many low‐ and middle‐income countries, including sub‐Saharan Africa (SSA), where access to care, workforce capacity, and mental health infrastructure remain limited.

**Objective:**

This study quantified the burden of mental and substance use disorders (SUDs) in SSA from 1990 to 2023 using Global Burden of Disease (GBD) 2023 estimates, with attention to long‐term trends, late‐period changes, country‐level heterogeneity, sex differences, and risk‐factor attribution.

**Methods:**

We analyzed GBD 2023 estimates for 46 SSA countries from 1990 to 2023, including prevalence, disability‐adjusted life years (DALYs), age‐standardized rates, sex‐specific estimates, country‐level burden, and selected risk factors. Percentage changes were calculated for 1990–2023, with sensitivity analyses for 1990–2019 and 2019–2023 and segmented log‐linear trend models using 2019 as the breakpoint.

**Results:**

From 1990 to 2023, the age‐standardized prevalence rate of mental disorders increased from 11,138.8 to 14,841.0 per 100,000 (+33.24%), while the age‐standardized DALY rate increased from 1634.9 to 2218.9 per 100,000 (+35.73%). Anxiety disorders showed the largest relative increases in age‐standardized prevalence (+90.99%) and DALY rates (+92.11%). Depressive disorders also increased, while ADHD age‐standardized rates remained largely stable. SUDs showed rising absolute burden despite declining age‐standardized prevalence and DALY rates. Late‐period analyses showed larger increases after 2019, particularly for anxiety disorders. Females had higher age‐standardized rates and larger relative increases for anxiety and depressive disorders, whereas males had higher rates of ADHD and SUDs. Among selected risk factors, sexual violence against children had the highest age‐standardized attributable DALY rate in 2023, while bullying victimization and intimate partner violence showed the largest relative increases.

**Conclusion:**

Mental and SUDs represent a growing public health challenge in SSA. Strengthening mental health systems, integrating services into primary and general healthcare platforms, expanding SUD prevention and treatment, addressing violence‐related risk factors, and reducing stigma are essential to improving mental health outcomes across the region.

## 1. Introduction

Mental disorders are among the leading causes of health‐related disability worldwide. According to the Global Burden of Disease (GBD) 2019 study, these conditions accounted for approximately 4.9% of global disability‐adjusted life years (DALYs), emphasizing their persistent impact on population health over the past three decades [1]. Despite their magnitude, mental disorders often receive limited attention in global health agendas, in part because of competing priorities such as infectious diseases, maternal and child health, and undernutrition in low‐ and middle‐income countries (LMICs). A large proportion of individuals with mental disorders do not receive treatment [2], particularly in LMICs, with treatment gaps exceeding 50% globally and reaching even higher levels in resource‐limited settings [3, 4].

Nowhere is this gap more pronounced than in sub‐Saharan Africa (SSA)—a region marked by rapid demographic transitions, fragile health systems, and limited access to mental health services [5, 6]. With one of the youngest and fastest‐growing populations globally, SSA faces mounting pressures from urbanization, unemployment, political instability, displacement, and infectious disease outbreaks—all known risk factors for poor mental health [7, 8]. Yet, national mental health systems across the region remain underdeveloped, with severe shortages in trained personnel, funding, and service coverage [7].

Cultural factors further complicate the landscape. Several studies show that in many parts of SSA, mental illness is often interpreted through spiritual or supernatural frameworks, leading to delayed care‐seeking and stigma [9, 10]. Such beliefs can reinforce harmful stereotypes and prevent individuals from accessing evidence‐based interventions. Meanwhile, the needs of adolescents and youth—a demographic disproportionately affected by mental health disorders—are particularly underaddressed. A global systematic review found that adolescents in SSA face high rates of depression, anxiety, and trauma‐related disorders, but rarely receive timely diagnosis or treatment [8]. Substance use disorders (SUDs) further compound this challenge and are particularly relevant among adolescents and young adults in SSA. Research indicates that over 40% of adolescents report some form of substance use, with alcohol and tobacco most commonly involved, followed by rising cannabis use [11–13]. These patterns are strongly associated with socioeconomic vulnerabilities such as poverty and unemployment, and SUDs frequently co‐occur with other mental health conditions, amplifying disability and care needs [14, 15].

Despite these challenges, empirical data on mental health burden in SSA remain scarce. Most existing studies are localized, often limited to urban centers or specific population groups, and vary widely in design and measurement [9, 16]. Many reviews also point to a lack of longitudinal data, regional representativeness, and standardized outcome metrics [7, 8]. As a result, policymakers and public health officials are left without the robust evidence needed to plan and scale effective mental health interventions.

The GBD dataset offers a useful framework for addressing part of this evidence gap. The GBD framework synthesizes data from multiple sources, including surveys, administrative data, published studies, and clinical records, to generate comparable estimates of disease burden across time, age, sex, and location. Its inclusion of mental and SUDs as major causes of nonfatal disease burden makes it valuable for assessing long‐term trends, identifying high‐burden populations, and comparing patterns across countries. Previous GBD‐based studies have provided important insights into the global and regional burden of mental disorders [1].

However, SSA‐specific analyses using the most recent GBD 2023 estimates remain important for understanding how mental and SUD burden has evolved across the region, particularly in the context of rapid demographic change, persistent treatment gaps, and late‐period disruptions. This study analyzed GBD 1990–2023 estimates for mental and SUDs across 46 SSA countries, with attention to prevalence, DALYs, age‐standardized rates (ASRs), sex differences, country‐level variation, selected risk factors, and late‐period trends. By providing a region‐focused update using GBD 2023 estimates, this study aims to inform future research, health‐system planning, and policy development for mental health in SSA.

## 2. Methods

### 2.1. Data Sources

We used estimates from the GBD 2023 study, an international collaborative initiative coordinated by the Institute for Health Metrics and Evaluation (IHME) at the University of Washington [17]. The GBD framework systematically quantifies the impact of diseases, injuries, and risk factors on population health across all countries and territories. The 2023 iteration provides annual estimates from 1990 through 2023 for 375 diseases and injuries and 88 risk factors, with data available for both sexes and all age groups in 204 countries and territories.

GBD estimates are derived from multiple sources, including population censuses, household surveys, disease registries, health facility records, and vital registration systems. Statistical modeling approaches are employed to account for incomplete or inconsistent reporting and to generate internally comparable estimates over time. For this study, we included data spanning both prepandemic and postpandemic periods to capture recent temporal patterns, including those occurring during the COVID‐19 era, which has been shown to substantially impact mental health outcomes globally [18], while also preserving a long‐term perspective on trends in SSA.

All GBD 2023 estimates included in this study were downloaded from the Global Health Data Exchange (GHDx) (https://ghdx.healthdata.org/series/global-burden-disease-gbd) [17].

### 2.2. Measures

#### 2.2.1. Mental Disorders

We included all mental disorders defined within the GBD 2023 cause list (Table 1). These comprised (1) major depressive disorder; (2) dysthymia; (3) anxiety disorders; (4) schizophrenia; (5) bipolar disorder; (6) anorexia nervosa; (7) bulimia nervosa; (8) conduct disorder; (9) attention‐deficit/hyperactivity disorder (ADHD); (10) autism spectrum disorder; (11) other mental disorders (including aggregated personality disorders); and (12) idiopathic developmental intellectual disability. Case definitions in the GBD study are based on the diagnostic criteria outlined in the International Classification of Diseases, Tenth Revision (ICD‐10), and the Diagnostic and Statistical Manual of Mental Disorders, Fourth Edition, Text Revision (DSM‐IV‐TR) [19, 20].

**TABLE 1:** Mental disorders and substance use disorder categories included in the GBD 2023 study, with diagnostic framing based on ICD‐10 and DSM‐IV‐TR criteria.

Disorder	Description/Common features
Major depressive disorder	Persistent sadness, loss of interest, low energy, and impaired daily functioning
Dysthymia (persistent depressive disorder)	Chronic, less severe form of depression lasting 2 years or more
Anxiety disorders	Excessive fear, worry, or nervousness that interferes with daily life (includes generalized anxiety, panic, phobias)
Schizophrenia	Severe mental disorder with symptoms such as hallucinations, delusions, and disorganized thinking
Bipolar disorder	Episodes of depression and mania/hypomania, involving mood swings and changes in activity/energy
Anorexia nervosa	Eating disorder characterized by weight loss, fear of gaining weight, and distorted body image
Bulimia nervosa	Eating disorder marked by binge eating followed by compensatory behaviors such as vomiting or excessive exercise
Conduct disorder	Repetitive pattern of antisocial, aggressive, or defiant behavior in children/adolescents
Attention‐deficit/hyperactivity disorder (ADHD)	Persistent inattention, hyperactivity, and impulsivity beginning in childhood
Autism spectrum disorder (ASD)	Developmental condition involving difficulties in social communication and restricted/repetitive behaviors
Other mental disorders (incl. personality disorders)	Includes a range of conditions such as borderline and antisocial personality disorders
Idiopathic developmental intellectual disability	Impaired intellectual functioning and adaptive behaviors with onset during childhood, cause unspecified
Alcohol use disorder	Harmful pattern of alcohol consumption leading to health problems, dependence, or impaired functioning
Drug use disorders, including opioid, cannabis, cocaine, amphetamine, and other drug use disorders	Problematic use of substances such as cannabis, opioids, or stimulants, often leading to dependence and health complications

*Note:* Diagnostic descriptions are summarized in alignment with GBD case definitions, which are based on ICD‐10 and DSM‐IV‐TR diagnostic criteria. These descriptions are intended as concise summaries rather than full diagnostic criteria.

#### 2.2.2. Substance Use Disorders

Given their close epidemiological and clinical links to mental disorders, we also examined alcohol use disorders and drug use disorders. In the GBD framework, alcohol use disorders and drug use disorders are reported as distinct cause categories; in this manuscript, “substance use disorders” refers to the broader category encompassing both. The latter category includes amphetamine, cannabis, opioid, and cocaine use disorders, as well as a residual grouping of other psychoactive SUDs.

#### 2.2.3. SSA Regional Definition

Countries were classified according to the SSA regional grouping used by the GBD 2023 study, which comprises 46 countries spanning four subregions: Eastern, Western, Central, and Southern Africa.

### 2.3. Data Analysis

The GBD employs standardized modeling strategies to generate estimates, including the Cause of Death Ensemble Model (CODEm), spatiotemporal Gaussian process regression (ST‐GPR), and the Bayesian meta‐regression tool DisMod‐MR. These approaches integrate diverse data sources, adjust for measurement error, and propagate uncertainty throughout the analytical process. Full methodological details are described elsewhere in the GBD literature [21].

For this analysis, we extracted prevalence and DALYs for all included mental and SUDs in SSA from 1990 to 2023. DALYs, a composite metric of health loss, are calculated as the sum of years of life lost (YLLs) due to premature mortality and years lived with disability (YLDs). We extracted ASRs directly from the GBD 2023 estimates, rather than calculating them independently, to facilitate valid comparisons over time and between populations.

We calculated the percentage change in ASRs between 1990 and 2023 using the formula as follows:

(1) % change=20231990 Rate− Rate1990 Rate×100

Additional analyses included risk‐factor attribution. Risk factors analyzed for their association with mental disorders in SSA included bullying victimization, sexual violence against children, intimate partner violence (IPV), and lead exposure. These exposures are estimated in GBD using population‐based surveys, surveillance data, and geospatial modeling.

In addition to the primary 1990–2023 percentage‐change analyses, we conducted sensitivity analyses excluding the post‐2019 period to assess whether overall trends were influenced by late‐period changes. Specifically, we recalculated percentage changes in age‐standardized prevalence and DALY rates from 1990 to 2019 and compared these with changes from 2019 to 2023. To further summarize temporal patterns, we fitted segmented log‐linear trend models with 2019 as the breakpoint and estimated annualized percentage changes before and after 2019. These analyses were intended to characterize changes in modeled GBD estimates over time and were not designed to attribute observed changes causally to the COVID‐19 pandemic or to changes in GBD modeling assumptions.

For country‐level analyses, we separately examined all‐age DALY numbers and age‐standardized DALY rates for mental disorders in 1990 and 2023. This approach was used to distinguish changes in absolute population‐level burden from changes in underlying rate‐level burden.

All statistical analyses and data visualizations were performed using *R* software (Version *R* 4.4.3) [22].

This study was reported in accordance with the Strengthening the Reporting of Observational Studies in Epidemiology (STROBE) reporting guideline; the completed STROBE checklist is provided as Supporting File 1.

## 3. Results

### 3.1. Trends in Disease Burden (DALYs) and Prevalence of Mental Disorders (1990–2023)

#### 3.1.1. Prevalence of Mental Disorders

**FIGURE 1:**
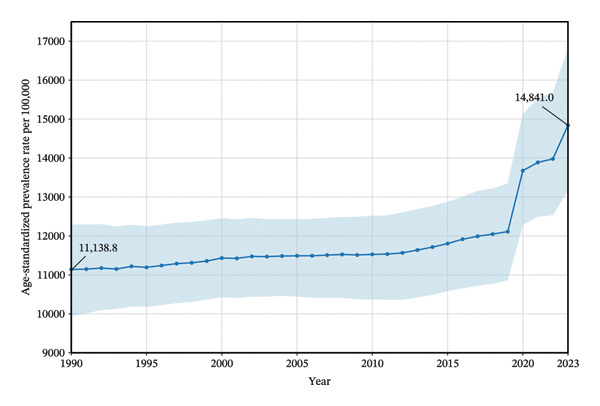
Trends in the age‐standardized prevalence rate of mental disorders overall in sub‐Saharan Africa, 1990–2023.

Figure 1 depicts the trends in age‐standardized prevalence rates of mental disorders from 1990 to 2023. The age‐standardized prevalence rate of mental disorders across SSA increased from 11,138.8 per 100,000 in 1990 to 14,841.0 per 100,000 in 2023, corresponding to a 33.24% increase.

#### 3.1.2. Prevalence by Disorder

**FIGURE 2:**
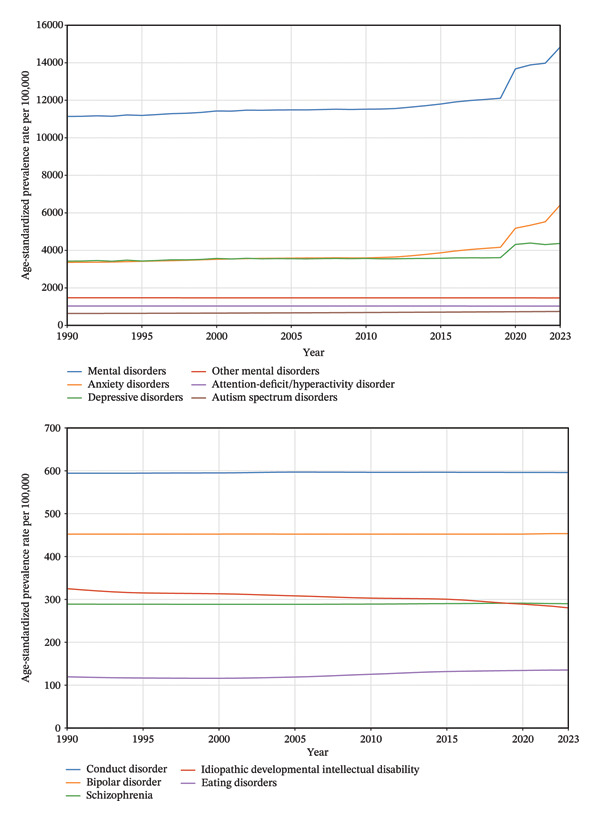
Trends in age‐standardized prevalence rates by mental disorder category in sub‐Saharan Africa, 1990–2023.

As shown in Figure 2 and Table 2, anxiety disorders showed the largest increase in age‐standardized prevalence rate, rising from 3360.0 to 6417.2 per 100,000 between 1990 and 2023 (+90.99%). Depressive disorders also increased from 3423.7 to 4369.8 per 100,000 (+27.63%). In contrast, ADHD age‐standardized prevalence remained essentially stable, changing from 1032.2 to 1028.7 per 100,000 (−0.35%).

**TABLE 2:** Age‐standardized prevalence rates for mental and substance use disorders in sub‐Saharan Africa by sex, 1990 and 2023.

Disorder/category	Sex	1990 rate (95% UI)	2023 rate (95% UI)	% Change
Mental disorders	Both	11,138.8 (9940.4–12,290.0)	14,841.0 (13,174.6–16,817.1)	33.24%
	Female	11,042.6 (9795.3–12,298.1)	15,340.6 (13,399.4–17,745.8)	38.92%
	Male	11,230.8 (10,127.1–12,438.8)	14,293.6 (12,790.7–16,050.8)	27.27%

Anxiety disorders	Both	3360.0 (2727.6–4185.5)	6417.2 (4936.1–8358.0)	90.99%
	Female	3845.2 (3104.9–4823.1)	7416.6 (5767.8–9817.5)	92.88%
	Male	2860.7 (2332.8–3530.2)	5375.5 (4068.3–6913.1)	87.91%

Depressive disorders	Both	3423.7 (2761.4–4157.6)	4369.8 (3638.5–5303.2)	27.63%
	Female	3916.1 (3153.8–4743.2)	4998.7 (4170.6–6063.1)	27.64%
	Male	2915.4 (2355.2–3559.9)	3691.2 (3060.7–4482.7)	26.61%

Schizophrenia	Both	289.1 (233.7–349.0)	290.2 (234.3–350.7)	0.39%
	Female	291.0 (234.2–351.5)	292.9 (236.7–355.6)	0.68%
	Male	287.2 (233.1–347.4)	287.2 (232.3–347.8)	0.00%

Bipolar disorder	Both	452.3 (376.4–551.8)	453.5 (377.1–554.8)	0.27%
	Female	464.0 (385.8–565.5)	465.1 (386.7–567.2)	0.25%
	Male	440.2 (366.7–538.1)	440.9 (366.8–540.0)	0.17%

Eating disorders	Both	119.5 (91.9–159.8)	135.3 (103.6–180.7)	13.23%
	Female	167.9 (130.4–220.3)	189.6 (147.4–248.0)	12.95%
	Male	68.9 (51.5–96.4)	78.3 (58.0–107.9)	13.64%

Conduct disorder	Both	594.3 (449.4–744.3)	596.1 (448.6–745.7)	0.31%
	Female	425.1 (298.8–564.9)	427.0 (299.7–558.6)	0.45%
	Male	766.4 (598.1–958.3)	764.7 (593.5–960.2)	−0.21%

Attention‐deficit/hyperactivity disorder	Both	1032.2 (719.5–1416.8)	1028.7 (716.9–1404.4)	−0.35%
	Female	620.0 (429.8–866.0)	620.8 (428.8–864.6)	0.13%
	Male	1456.3 (1019.7–2006.3)	1446.6 (1015.5–1979.0)	−0.66%

Autism spectrum disorders	Both	635.4 (268.1–1454.4)	744.1 (360.6–1383.6)	17.09%
	Female	347.4 (146.2–798.8)	408.8 (197.1–764.7)	17.65%
	Male	928.8 (391.7–2122.4)	1089.5 (530.8–2028.1)	17.30%

Idiopathic developmental intellectual disability	Both	325.1 (64.1–749.4)	280.4 (70.5–638.5)	−13.76%
	Female	309.5 (73.6–678.9)	274.8 (77.8–577.1)	−11.19%
	Male	341.1 (53.0–823.8)	286.8 (56.4–711.2)	−15.91%

Other mental disorders	Both	1472.6 (1161.5–1830.5)	1466.4 (1154.8–1822.2)	−0.42%
	Female	1197.9 (949.0–1529.0)	1197.9 (949.4–1528.2)	−0.00%
	Male	1755.8 (1375.1–2204.8)	1758.9 (1377.2–2205.4)	0.18%

Substance use disorders	Both	1411.7 (1231.3–1599.1)	1284.7 (1140.3–1442.6)	−8.99%
	Female	761.0 (664.6–865.6)	720.5 (634.7–809.9)	−5.32%
	Male	2076.4 (1804.3–2369.5)	1890.5 (1660.4–2137.7)	−8.95%

Alcohol use disorders	Both	1095.8 (931.6–1279.7)	984.9 (851.5–1141.5)	−10.12%
	Female	503.8 (420.4–597.6)	484.8 (410.0–573.4)	−3.77%
	Male	1698.5 (1439.8–1973.3)	1522.6 (1313.5–1771.5)	−10.36%

Drug use disorders	Both	322.1 (264.5–389.0)	305.1 (249.0–371.6)	−5.29%
	Female	259.0 (218.4–301.9)	237.3 (198.5–278.6)	−8.39%
	Male	388.7 (312.5–478.5)	377.1 (301.0–465.2)	−2.99%

#### 3.1.3. DALYs for Mental Disorders

**FIGURE 3:**
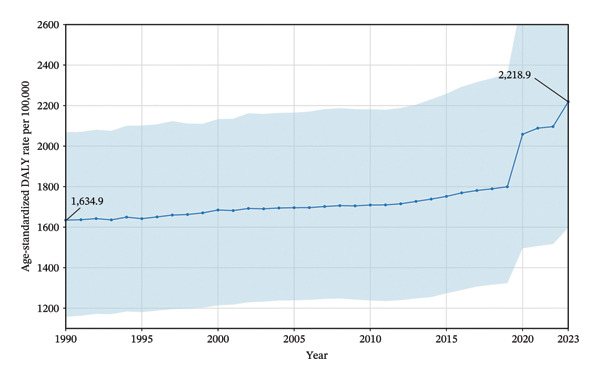
Trends in age‐standardized DALY rates of mental disorders in sub‐Saharan Africa, 1990–2023.

Figure 3 and Table 3 show the trends in age‐standardized DALYs for mental disorders across SSA from 1990 to 2023. The age‐standardized DALY rate for mental disorders increased from 1634.9 per 100,000 in 1990 to 2218.9 per 100,000 in 2023, corresponding to a 35.73% increase.

**TABLE 3:** Age‐standardized DALY rates for mental and substance use disorders in sub‐Saharan Africa by sex, 1990 and 2023.

Disorder/category	Sex	1990 rate (95% UI)	2023 rate (95% UI)	% Change
Mental disorders	Both	1634.9 (1158.4–2069.0)	2218.9 (1598.3–2972.4)	35.73%
	Female	1665.2 (1162.9–2142.1)	2327.1 (1690.5–3193.2)	39.75%
	Male	1602.4 (1145.3–2043.6)	2101.6 (1540.4–2808.4)	31.15%

Anxiety disorders	Both	394.8 (254.8–551.0)	758.4 (485.2–1129.0)	92.11%
	Female	449.6 (288.0–629.9)	871.8 (559.4–1321.9)	93.91%
	Male	338.4 (220.2–479.3)	640.6 (410.0–942.4)	89.30%

Depressive disorders	Both	579.0 (382.9–833.7)	773.8 (510.7–1121.0)	33.64%
	Female	658.7 (437.0–947.4)	880.5 (583.7–1271.5)	33.67%
	Male	496.7 (326.8–715.7)	658.9 (432.0–958.9)	32.65%

Schizophrenia	Both	181.1 (128.0–235.6)	182.7 (130.4–237.7)	0.84%
	Female	180.0 (127.2–232.7)	181.9 (130.0–235.0)	1.02%
	Male	182.3 (128.9–237.5)	183.5 (130.5–237.5)	0.66%

Bipolar disorder	Both	96.1 (60.1–137.0)	96.8 (60.6–138.3)	0.70%
	Female	97.6 (61.3–138.9)	98.2 (61.5–139.8)	0.56%
	Male	94.5 (59.2–134.9)	95.3 (59.5–136.6)	0.77%

Eating disorders	Both	25.1 (14.6–36.7)	28.4 (16.6–42.1)	13.49%
	Female	35.1 (20.6–51.0)	39.6 (23.2–57.6)	13.09%
	Male	14.6 (8.3–22.0)	16.7 (9.6–25.5)	14.28%

Conduct disorder	Both	71.6 (40.7–115.6)	72.2 (41.0–115.5)	0.78%
	Female	51.0 (28.2–83.3)	51.4 (28.4–84.1)	0.76%
	Male	92.5 (53.9–149.1)	92.9 (54.0–148.9)	0.34%

Attention‐deficit/hyperactivity disorder	Both	44.3 (25.7–67.6)	44.3 (25.8–66.7)	0.05%
	Female	26.4 (15.0–39.8)	26.6 (15.1–39.9)	0.52%
	Male	62.6 (36.6–96.0)	62.4 (36.6–95.1)	−0.27%

Autism spectrum disorders	Both	118.2 (46.3–254.0)	140.5 (62.2–308.2)	18.83%
	Female	64.3 (24.9–138.5)	76.6 (33.8–168.8)	19.28%
	Male	173.2 (68.0–371.6)	206.1 (91.6–447.2)	19.04%

Idiopathic developmental intellectual disability	Both	16.8 (4.0–40.1)	14.0 (3.9–33.0)	−16.84%
	Female	15.7 (4.6–35.1)	13.4 (4.3–29.8)	−14.45%
	Male	18.0 (3.2–45.2)	14.7 (3.6–35.5)	−18.68%

Other mental disorders	Both	107.9 (71.6–153.4)	107.9 (71.9–153.8)	0.06%
	Female	86.8 (57.2–123.5)	87.0 (57.7–124.3)	0.31%
	Male	129.6 (84.5–183.0)	130.7 (85.5–184.6)	0.82%

Substance use disorders	Both	250.2 (195.9–313.0)	236.9 (188.2–297.1)	−5.32%
	Female	143.2 (108.9–178.3)	145.5 (113.0–180.7)	1.60%
	Male	358.5 (272.9–458.6)	335.4 (250.3–443.0)	−6.43%

Alcohol use disorders	Both	181.3 (134.1–236.7)	166.8 (121.6–216.0)	−8.00%
	Female	81.2 (58.4–107.4)	85.7 (65.1–111.8)	5.55%
	Male	282.1 (200.1–384.1)	254.0 (177.1–346.2)	−9.94%

Drug use disorders	Both	69.0 (50.2–90.2)	70.1 (52.6–91.1)	1.73%
	Female	62.0 (45.6–82.2)	59.8 (45.1–77.2)	−3.56%
	Male	76.4 (53.7–103.9)	81.4 (58.7–114.2)	6.56%

Abbreviations: ASDR, age‐standardized DALY rate; %, percentage change, as outlined in formula 1 in the Methods section.

#### 3.1.4. DALYs by Disorder

**FIGURE 4:**
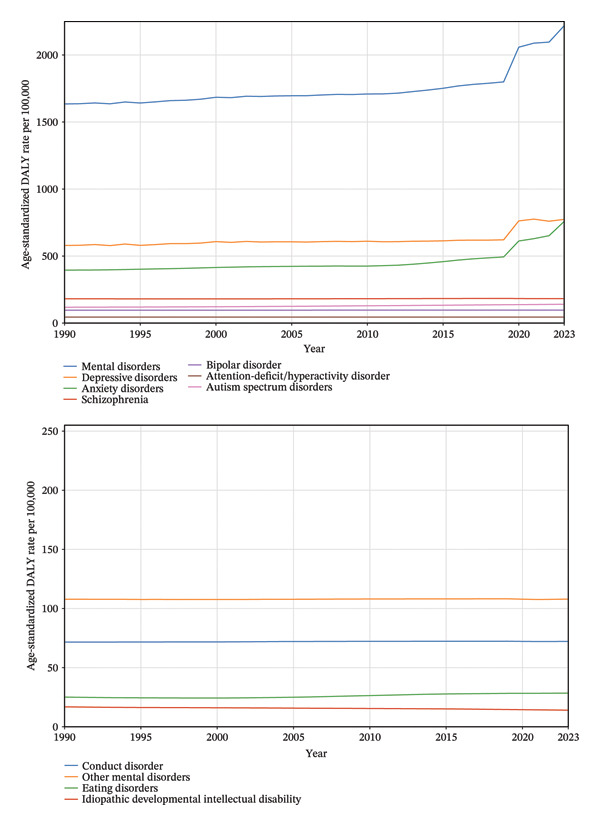
Trends in DALY rates, age‐standardized, of mental disorders in the SSA region (1990–2023).

Figure 4 shows trends in age‐standardized DALYs by mental disorders across SSA from 1990 to 2023. Anxiety disorders demonstrated the largest relative increase in age‐standardized DALY rate, rising from 394.8 to 758.4 per 100,000 between 1990 and 2023 (+92.11%). Depressive disorders also increased from 579.0 to 773.8 per 100,000 (+33.64%). ADHD showed little change in age‐standardized DALY rate, remaining approximately stable from 44.3 to 44.3 per 100,000 (+0.05%).

#### 3.1.5. SUDs

Between 1990 and 2023, the absolute all‐age DALY burden attributable to SUDs increased from 887,210 to 2,213,977 (+149.54%). However, the age‐standardized DALY rate declined from 250.2 to 236.9 per 100,000 (−5.32%; Table 3). Similarly, all‐age prevalent cases increased from 5,357,222 to 12,977,211 (+142.24%), while the age‐standardized prevalence rate declined from 1411.7 to 1284.7 per 100,000 (−8.99%; Table 2).

Alcohol use disorders accounted for the largest share of substance‐related DALYs. All‐age DALYs increased from 611,797 to 1,479,723 (+141.86%), while the age‐standardized DALY rate declined from 181.3 to 166.8 per 100,000 (−8.00%). Drug use disorders increased from 275,412 to 734,254 all‐age DALYs (+166.60%), while the age‐standardized DALY rate changed only modestly, from 69.0 to 70.1 per 100,000 (+1.73%).

#### 3.1.6. Sensitivity and Late‐Period Trend Analyses

Sensitivity analyses examining late‐period changes are shown in Table 4. Sensitivity analyses excluding 2020–2023 showed that increases in mental disorder burden were smaller when the analysis was restricted to 1990–2019. The age‐standardized prevalence rate for mental disorders increased by 8.72% from 1990 to 2019, compared with 33.24% from 1990 to 2023. Similarly, the age‐standardized DALY rate increased by 10.07% from 1990 to 2019, compared with 35.73% from 1990 to 2023. Between 2019 and 2023, the age‐standardized prevalence and DALY rates for mental disorders increased by 22.55% and 23.31%, respectively.

**TABLE 4:** Sensitivity and segmented trend analyses of age‐standardized prevalence and DALY rates in sub‐Saharan Africa, 1990–2023.

Outcome	1990–2023% change	1990–2019% change	2019–2023% change	Segmented APC before 2019	Segmented APC after 2019	Slope change *p* value
Mental disorder prevalence ASR	33.24%	8.72%	22.55%	0.25%/yr	2.55%/yr	1.38e‐06
Anxiety disorder prevalence ASR	90.99%	23.94%	54.10%	0.62%/yr	7.00%/yr	6.04e‐06
Depressive disorder prevalence ASR	27.63%	5.50%	20.98%	0.17%/yr	0.19%/yr	0.958
Attention‐deficit/hyperactivity disorder prevalence ASR	−0.35%	−0.23%	−0.12%	−0.00%/yr	−0.03%/yr	0.599
Substance use disorder prevalence ASR	−8.99%	−5.30%	−3.90%	−0.13%/yr	−1.21%/yr	0.00774
Alcohol use disorder prevalence ASR	−10.12%	−5.03%	−5.36%	−0.10%/yr	−1.71%/yr	0.00508
Drug use disorder prevalence ASR	−5.29%	−6.34%	1.13%	−0.23%/yr	0.47%/yr	0.267
Mental disorder DALY ASDR	35.73%	10.07%	23.31%	0.30%/yr	2.32%/yr	6.41e‐06
Anxiety disorder DALY ASDR	92.11%	24.95%	53.75%	0.66%/yr	7.00%/yr	6.67e‐06
Depressive disorder DALY ASDR	33.64%	7.26%	24.59%	0.23%/yr	0.25%/yr	0.961
Attention‐deficit/hyperactivity disorder DALY ASDR	0.05%	0.26%	−0.21%	0.01%/yr	−0.02%/yr	0.383
Substance use disorder DALY ASDR	−5.32%	−7.28%	2.11%	−0.22%/yr	0.99%/yr	0.103
Alcohol use disorder DALY ASDR	−8.00%	−8.22%	0.24%	−0.21%/yr	0.35%/yr	0.52
Drug use disorder DALY ASDR	1.73%	−4.80%	6.87%	−0.26%/yr	2.58%/yr	2.85e‐05

This late‐period pattern was particularly pronounced for anxiety disorders. Anxiety disorder age‐standardized prevalence increased by 23.94% from 1990 to 2019 and by 54.10% from 2019 to 2023, while the age‐standardized DALY rate increased by 24.95% from 1990 to 2019 and by 53.75% from 2019 to 2023. Depressive disorders also increased, although the late‐period change was less pronounced than for anxiety disorders, with age‐standardized prevalence increasing by 5.50% from 1990 to 2019 and by 20.98% from 2019 to 2023, and age‐standardized DALY rates increasing by 7.26% and 24.59%, respectively. ADHD ASRs remained stable across both periods.

Segmented log‐linear trend models were consistent with these findings. For mental disorders overall, the annualized increase in age‐standardized prevalence rate was 0.25% per year before 2019 and 2.55% per year after 2019. The corresponding annualized changes in age‐standardized DALY rates were 0.30% per year before 2019 and 2.32% per year after 2019. Anxiety disorders showed the clearest late‐period acceleration, with annualized increases of approximately 0.62%–0.66% per year before 2019 and approximately 7.00% per year after 2019.

### 3.2. Country‐Specific Trends

**FIGURE 5:**
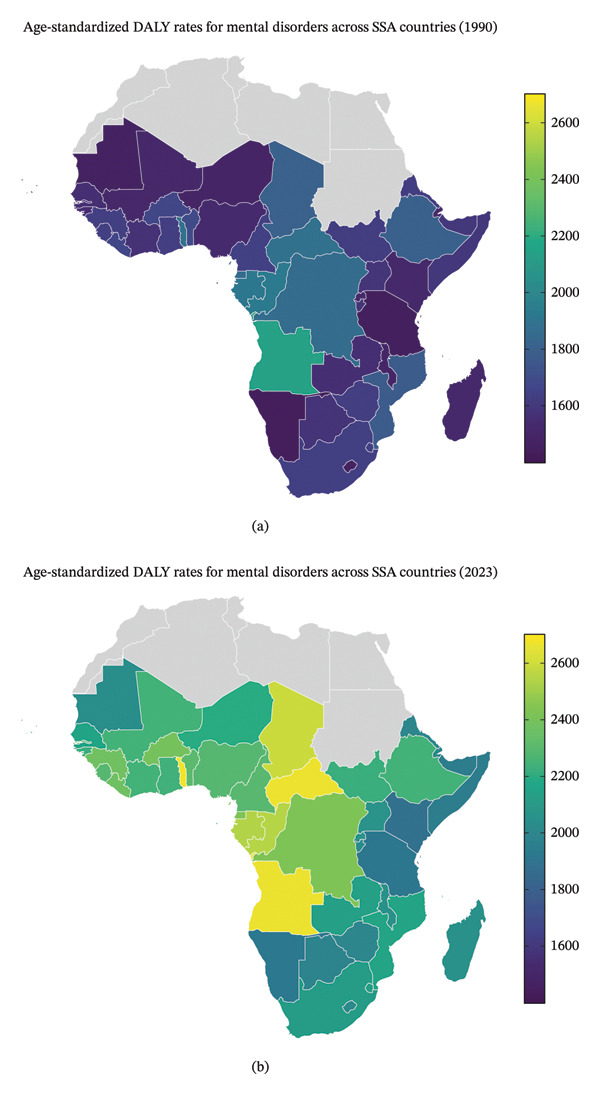
(a) Age‐standardized DALYs across SSA countries (1990) and (b) age‐standardized DALYs across SSA countries (2023).

Figures 5A,B illustrate the country‐specific distribution of age‐standardized DALY rates attributable to mental disorders in 1990 and 2023, while Supporting Tables 1 and 2 provide detailed country‐level all‐age DALY and age‐standardized DALY rate estimates. Across SSA, the absolute burden varied substantially by country, with the largest total DALY burdens in 2023 observed in Nigeria, Ethiopia, the Democratic Republic of the Congo, South Africa, and Tanzania. Nigeria had the highest all‐age DALY burden in 2023, with 5,377,571 DALYs attributable to mental disorders, followed by Ethiopia with 2,365,758 DALYs and the Democratic Republic of the Congo with 2,071,443 DALYs.

Absolute all‐age DALYs increased across all 46 SSA countries between 1990 and 2023. Age‐standardized DALY rates also increased across countries, although the magnitude of increase varied. The largest relative increases in age‐standardized DALY rates were observed in Nigeria (+49.4%), Mali (+48.6%), Niger (+47.7%), Gambia (+46.1%), and Guinea (+45.4%).

Countries with the largest absolute DALY burdens were not always the same countries with the largest relative increases in age‐standardized DALY rates. For example, Ethiopia, the Democratic Republic of the Congo, South Africa, and Tanzania ranked among the highest‐burden countries by total DALYs in 2023, while several West African countries showed larger relative increases in age‐standardized DALY rates.

### 3.3. Gender Differences in DALYs and Prevalence

#### 3.3.1. Prevalence by Gender

As shown in Table 2, age‐standardized prevalence rates for mental disorders increased in both females and males between 1990 and 2023. Among females, the age‐standardized prevalence rate increased from 11,042.6 per 100,000 in 1990 to 15,340.6 per 100,000 in 2023, corresponding to a 38.92% increase. Among males, the rate increased from 11,230.8 to 14,293.6 per 100,000, corresponding to a 27.27% increase.

#### 3.3.2. Prevalence by Disorder and Gender

Anxiety disorders increased substantially in both sexes. Among females, the age‐standardized prevalence rate increased from 3845.2 to 7416.6 per 100,000 between 1990 and 2023, corresponding to a 92.88% increase. Among males, the rate increased from 2860.7 to 5375.5 per 100,000, corresponding to an 87.91% increase. Depressive disorders also increased in both sexes, from 3916.1 to 4998.7 per 100,000 among females (+27.64%) and from 2915.4 to 3691.2 per 100,000 among males (+26.61%). In contrast, ADHD age‐standardized prevalence rates were largely stable, changing from 620.0 to 620.8 per 100,000 among females (+0.13%) and from 1456.3 to 1446.6 per 100,000 among males (−0.66%).

#### 3.3.3. DALYs by Gender

As shown in Table 3, the gender distribution of mental disorder DALYs shows notable differences across SSA. Age‐standardized DALY rates for mental disorders increased in both sexes. Among females, the age‐standardized DALY rate increased from 1665.2 per 100,000 in 1990 to 2327.1 per 100,000 in 2023, corresponding to a 39.75% increase. Among males, the rate increased from 1602.4 to 2101.6 per 100,000, corresponding to a 31.15% increase.

#### 3.3.4. DALYs by Disorder and Gender

For anxiety disorders, age‐standardized DALY rates increased from 449.6 to 871.8 per 100,000 among females (+93.91%) and from 338.4 to 640.6 per 100,000 among males (+89.30%). Depressive disorder DALY rates also increased in both sexes, from 658.7 to 880.5 per 100,000 among females (+33.67%) and from 496.7 to 658.9 per 100,000 among males (+32.65%). ADHD age‐standardized DALY rates were largely stable, changing from 26.4 to 26.6 per 100,000 among females (+0.52%) and from 62.6 to 62.4 per 100,000 among males (−0.27%). For substance‐use disorders, sex‐specific trends differed from those observed for mental disorders overall. Among females, the age‐standardized DALY rate changed modestly from 143.2 to 145.5 per 100,000 (+1.60%), while among males, it declined from 358.5 to 335.4 per 100,000 (−6.43%). Despite this decline, males continued to have substantially higher age‐standardized DALY rates for SUDs than females in 2023.

Overall, females showed larger relative increases in age‐standardized prevalence and DALY rates for mental disorders, particularly anxiety and depressive disorders, while males continued to have higher ASRs for ADHD and SUDs.

### 3.4. Risk‐Factor Attribution to Mental Disorder Burden

**FIGURE 6:**
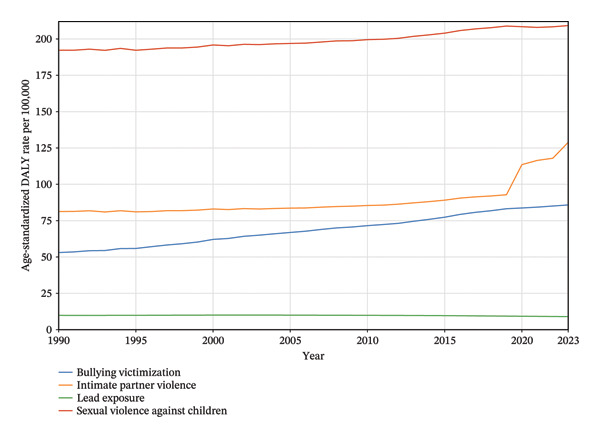
Trends of age‐standardized DALY rates attributable to key risk factors for mental disorders in SSA, 1990–2023.

Figure 6 presents the trends in age‐standardized DALY rates attributable to selected risk factors for mental disorders in SSA from 1990 to 2023. The risk factors included bullying victimization, sexual violence against children, IPV, and lead exposure. In 2023, sexual violence against children had the highest age‐standardized attributable DALY rate, at 209.2 per 100,000, followed by IPV at 128.9 per 100,000, bullying victimization at 85.8 per 100,000, and lead exposure at 9.0 per 100,000.

Between 1990 and 2023, age‐standardized attributable DALY rates changed differentially across risk factors. Bullying victimization showed the largest relative increase, rising from 53.0 to 85.8 per 100,000 (+61.74%). IPV also increased substantially, from 81.3 to 128.9 per 100,000 (+58.61%). Sexual violence against children increased more modestly, from 192.2 to 209.2 per 100,000 (+8.83%). In contrast, the age‐standardized DALY rate attributable to lead exposure declined slightly, from 9.8 to 9.0 per 100,000 (−8.76%).

**FIGURE 7:**
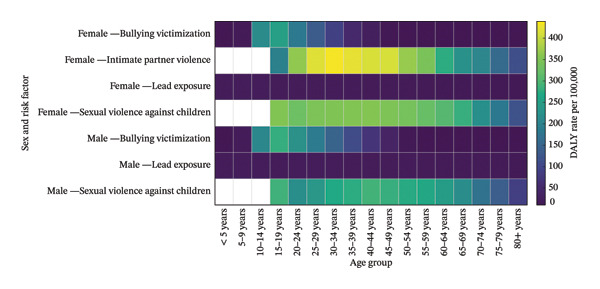
Age‐specific DALY rates attributable to selected risk factors for mental disorders in sub‐Saharan Africa by sex, 2023.

Figure 7 shows age‐specific DALY rates attributable to these risk factors by sex in 2023. Bullying victimization contributed most prominently during adolescence, peaking in the 15–19‐year age group in both females and males. IPV ‐attributable DALY rates were reported among females and were highest in young and middle adulthood, peaking around the 30–34‐year age group. Sexual violence against children contributed substantially across multiple adult age groups and was higher among females than males. Lead exposure accounted for a much smaller attributable DALY rate overall, with the highest rates observed in younger age groups.

## 4. Discussion

This study quantified the burden of mental and SUDs in SSA over a 33‐year period using GBD 2023 estimates. It provides a timely SSA‐focused analysis of mental and SUD burden using the most recent GBD 2023 estimates, incorporating sensitivity, segmented trend, country‐level, sex‐specific, and risk‐factor analyses. Our findings show that the burden of mental disorders in SSA increased substantially between 1990 and 2023, although patterns differed across disorders and metrics. Age‐standardized prevalence and DALY rates increased for mental disorders overall, with the largest relative increases observed for anxiety disorders. Depressive disorders also increased in both age‐standardized prevalence and DALY rates, while ADHD remained largely stable. SUDs showed a different pattern, with substantial increases in absolute all‐age prevalent cases and DALYs but declining age‐standardized prevalence and DALY rates, highlighting the importance of distinguishing demographic expansion from changes in underlying rate‐level burden.

These trends were not uniform across the full study period. Sensitivity analyses showed that increases in mental disorder burden were smaller when the analysis was restricted to 1990–2019, while a larger increase was observed from 2019 to 2023. Segmented log‐linear trend models similarly showed higher annualized increases after 2019, particularly for anxiety disorders. This late‐period acceleration may reflect a combination of pandemic‐era stressors, including social isolation, bereavement, economic insecurity, school disruption, health‐system strain, and reduced access to routine mental health services. However, these findings should be interpreted cautiously. Because the analysis was based on GBD 2023 modeled estimates, the observed late‐period rise may also reflect updated data incorporation, changes in estimation procedures, or other GBD modeling assumptions. Our analysis was not designed to causally attribute the rise to the COVID‐19 pandemic or to distinguish epidemiological change from modeling‐related effects.

Overall, our findings align with global evidence showing rising absolute burden of mental disorders over recent decades, while also underscoring the heterogeneity of age‐standardized trends across specific disorders and regions. Prior global and regional GBD analyses have documented increasing burden of common mental disorders, particularly anxiety and depression, with late‐period acceleration in several settings [1, 23–25]. In SSA, anxiety disorders showed the clearest increase, with both age‐standardized prevalence and DALY rates rising markedly. Depressive disorders also increased, although less sharply than anxiety disorders. In contrast, ADHD ASRs remained largely stable over the study period. These findings suggest that the growing mental health burden in SSA reflects both demographic expansion and disorder‐specific changes in ASRs, rather than a single uniform trend across all conditions.

Gender differences in mental health burden were prominent, with females in SSA exhibiting a higher burden for anxiety, depression, and eating disorders than their male counterparts. This mirrors patterns found globally, including in China, where females are disproportionately affected by anxiety and depression [1, 24]. Research consistently shows that females in SSA are more vulnerable to mental health issues due to various sociocultural, economic, and psychological factors. These include the pervasive issue of IPV, as well as socioeconomic constraints, which exacerbate mental health vulnerabilities [26, 27]. For instance, IPV has been strongly linked to depression among SSA women, with studies highlighting how childhood trauma and IPV contribute to long‐term mental health challenges [27, 28].

In contrast, males in SSA continued to have higher ASRs of ADHD, conduct disorder, and SUDs compared with females. However, ADHD age‐standardized prevalence and DALY rates remained largely stable between 1990 and 2023, suggesting that sex differences in ADHD burden should not be interpreted as evidence of a large rate‐level increase over time. ADHD is consistently reported more frequently among boys than girls, including in African and global systematic reviews, with male predominance often attributed to greater externalizing and hyperactive‐impulsive symptoms that are more visible to caregivers, teachers, and clinicians [29, 30]. In contrast, ADHD in girls may be more likely to present with inattentive symptoms and may therefore be under‐recognized. In SSA, persistent sex differences in ADHD estimates may therefore reflect a combination of true epidemiological differences, diagnostic practices, school‐based recognition, and underdiagnosis among girls, particularly in settings with limited child mental health services.

The divergent pattern observed for SUDs is also important for interpretation. Although all‐age SUD DALYs and prevalent cases increased substantially, age‐standardized DALY and prevalence rates declined modestly over the study period. This suggests that the growing absolute burden of SUDs in SSA is likely driven largely by population growth and changing age structure rather than a uniform increase in underlying ASRs. Nevertheless, the absolute expansion of SUD burden remains highly relevant for service planning, particularly given SSA’s youthful population and the concentration of substance use–related harms among adolescents and young adults.

Country‐level findings further emphasize the distinction between absolute burden and rate‐level change. Countries with the largest all‐age DALY burdens in 2023, including Nigeria, Ethiopia, the Democratic Republic of the Congo, South Africa, and Tanzania, were generally large‐population countries. In contrast, the largest relative increases in age‐standardized DALY rates were observed in Nigeria, Mali, Niger, Gambia, and Guinea. These patterns suggest that policy interpretation should not rely solely on total DALY counts, as high absolute burden may reflect population size, while ASRs provide additional insights into underlying rate‐level differences across countries.

Risk‐factor attribution analyses also highlighted important heterogeneity. In 2023, sexual violence against children had the highest age‐standardized attributable DALY rate among the selected risk factors, followed by IPV, bullying victimization, and lead exposure. From 1990 to 2023, the largest relative increases were observed for bullying victimization and IPV, whereas lead exposure declined slightly. Age‐specific analyses showed that bullying victimization contributed most prominently during adolescence, IPV‐attributable burden was reported among females and peaked in young to middle adulthood, and sexual violence against children contributed substantially across multiple age groups. These findings are consistent with broader evidence linking childhood adversity, interpersonal violence, and gender‐based violence with depression, anxiety, trauma‐related symptoms, substance use, and long‐term mental health vulnerability in SSA [27, 28, 31–37].

The observed increase in absolute mental health burden in SSA likely reflects a combination of demographic growth, changing age structure, and interconnected sociodemographic pressures. Rapid urbanization has already begun to transform the region’s mental health landscape, with studies from SSA linking urban poverty, informal settlements, overcrowding, socioeconomic stress, and reduced access to services with poorer mental health outcomes, including depressive symptoms [37]. While urban areas can provide better access to healthcare and social services, they also introduce significant challenges, such as overcrowded living conditions, inadequate infrastructure, and increased exposure to crime and violence. These conditions have been shown to exacerbate mental health issues, particularly in urban slums, which are characterized by poverty and poor living environments [38, 39]. The lack of sufficient healthcare facilities and mental health services in these urban areas further complicates the ability of residents to seek care, leading to a significant gap in meeting mental health needs [40]. Displaced populations, particularly refugees, face further psychological stress due to the loss of community ties, insecurity, and limited access to healthcare, with those in urban settings facing unique challenges compared to their counterparts in refugee camps [41, 42].

Taken together, these social and demographic pressures may help explain why the absolute burden of mental disorders increased substantially in SSA, even when age‐standardized trends varied by disorder. Population growth and a youthful age structure increase the number of individuals entering high‐risk age groups for mental and SUDs, while urbanization, displacement, violence, unemployment, and economic insecurity may increase exposure to chronic stressors that contribute to anxiety, trauma‐related symptoms, and substance use. At the same time, rising awareness and gradual improvements in recognition may have influenced the detection of some mental and neurodevelopmental conditions, although our findings suggest that ADHD ASRs remained largely stable over time. Thus, the observed GBD trends likely reflect a combination of demographic expansion, changing exposure to risk factors, and evolving detection rather than a single uniform epidemiological process.

These epidemiological pressures are compounded by the youth bulge in SSA, driven by high fertility rates, which adds another layer of complexity to the region’s mental health crisis. As Kabiru et al. note, the rapidly growing youth population demands significant attention to mental health needs, which are often overlooked in national health planning [43]. Young people, especially in urban and impoverished areas, face considerable socioeconomic pressures and a lack of mental health services, rendering them particularly vulnerable to mental health issues [44]. At the same time, the mental health infrastructure in SSA remains severely underdeveloped, contributing to a substantial treatment gap. The region faces a critical shortage of mental health professionals, with ratios often as low as 0.1 psychiatrists per 100,000 people [45], particularly in rural areas where specialized mental health services are virtually nonexistent. The lack of trained professionals and the absence of screening programs complicate the diagnosis and treatment of mental disorders, making it difficult to provide adequate care [14, 44]. Moreover, limited access to care, driven by factors such as geographical distance from health facilities, high travel costs, and financial constraints, constitutes another key barrier. Cultural perceptions of mental illness also contribute to the low utilization of formal healthcare services, with many individuals opting for traditional healing practices instead [44, 46].

A significant driver of the underdiagnosis and treatment gap in SSA is the stigma surrounding mental health, which remains a pervasive issue across the region. Societal attitudes often equate mental illness with weakness or moral failure, leading to discrimination and social isolation for those affected [47]. This stigma is particularly pronounced in communities with high levels of cultural misconception about mental health, which further discourages individuals from seeking professional care [48]. Additionally, many SSA countries suffer from low mental health literacy, among both the general population and healthcare providers, which contributes to widespread underdiagnosis and misdiagnosis of mental health disorders [49, 50]. The lack of mental health training for healthcare providers exacerbates this issue, as they often lack the confidence or skills to diagnose and treat mental health conditions effectively [49]. Furthermore, the focus of health research in SSA has historically been skewed toward more visible health issues, such as infectious diseases, while mental health remains significantly underrepresented [43]. This imbalance, coupled with historical neglect, has contributed to the underreporting and underestimation of mental health needs across the region, further perpetuating the treatment gap [46, 51].

### 4.1. Policy, Practice, and Public Health Implications

To address the growing mental health burden in SSA, integrating mental health into primary care and general healthcare systems is an essential strategy. In this context, **primary care** refers to first‐contact, community‐facing services delivered through clinics, health centers, and frontline health workers, while **general healthcare systems** refer more broadly to nonspecialist health services across outpatient care, maternal and child health, HIV care, emergency care, and community‐based platforms. This distinction is important because most individuals in SSA are unlikely to access specialized psychiatric services, which remain scarce, difficult to access, and often concentrated in urban centers or capital cities. Regional data further support this approach: The WHO African Region has a severely constrained mental health workforce, with approximately 0.1 psychiatrists per 100,000 population and major gaps in specialist service availability [52]. Given this limited specialist capacity, integration into primary and general healthcare platforms provides a pragmatic route to expand early detection, referral, basic psychosocial support, and continuity of care. The WHO Mental Health Gap Action Programme (mhGAP) supports this model by equipping nonspecialist health workers to identify and manage priority mental, neurological, and substance use conditions in routine care settings [53]. This integration is particularly relevant for high‐contact services such as maternal and child health, HIV care, adolescent health services, and outpatient primary care, where mental health conditions may otherwise remain undetected. By aligning mental health services with existing health delivery mechanisms, this approach can improve reach, reduce reliance on scarce specialists, and support more comprehensive care across the life course.

Moreover, given the acute shortage of mental health professionals in SSA, capacity building for mental health workers is critical. With many countries in the region facing significant deficits in trained personnel, policies should prioritize training programs tailored to local needs and also consider task‐shifting strategies, where less specialized healthcare workers are trained to manage mental health conditions [14, 54, 55]. Strengthening the workforce will not only expand access but also improve the competencies of healthcare providers, ensuring better recognition and management of mental health conditions.

In addition to workforce development, policy frameworks for mental health must be aligned with broader health initiatives and supported by sustainable funding mechanisms. External funding remains a significant source for health programming in SSA, but this often leads to inconsistent program implementation [56]. Establishing domestic resource allocation and financing models is crucial to building a resilient and equitable mental health system that can meet the needs of vulnerable populations across the region [57]. Furthermore, addressing the stigma surrounding mental illness is paramount to improving help‐seeking behaviors. Public awareness campaigns and community‐level mental health literacy initiatives can play a significant role in mitigating stigma, thereby increasing service utilization and reducing barriers to care [58].

Interventions are also urgently needed to address the rising absolute burden of SUDs, despite the modest decline in ASRs. Policies should include scaling up prevention programs targeting adolescents and young adults, strengthening community‐based rehabilitation and harm‐reduction services, and integrating SUD treatment into primary care. Without such measures, the growing absolute burden of SUDs will continue to undermine progress in mental health and public health more broadly.

Finally, to inform and guide these policy efforts, investing in mental health research is essential. Comprehensive data on mental health prevalence in SSA will provide the evidence necessary to shape effective policies, track progress, and allocate resources more efficiently [45]. Strengthening epidemiological research will be key in addressing the mental health crisis in SSA and ensuring that policy decisions are based on sound, context‐specific evidence.

### 4.2. Strengths and Limitations

This study benefits from the use of the standardized GBD 2023 methodology, which allows for comparability over time and between countries, offering a robust framework for understanding trends in mental disorders across different regions, including SSA. The use of this methodology ensures that findings are consistent and reliable, enabling direct comparisons of mental health burdens across multiple nations. Additionally, the inclusion of a wide range of mental disorders and SUDs in the analysis adds depth and comprehensiveness to the study, capturing the varied spectrum of mental health challenges in SSA. This broad approach provides a holistic view of the mental health landscape in the region, covering conditions such as anxiety, depression, ADHD, and substance use, which are often underreported or overlooked in more narrowly focused studies.

However, there are notable limitations to this study. One key limitation is the variability in data quality across different countries. As is common in global health assessments, the reliance on modeled estimates becomes necessary when primary data are sparse or unavailable, especially in regions like SSA where mental health data are often not systematically collected or reported [45]. Although the GBD methodology uses standardized statistical models to estimate disease burden and improve comparability across countries and time, these estimates remain subject to uncertainty due to the quality, availability, and representativeness of the underlying data. This is particularly relevant when interpreting the late‐period acceleration observed after 2019. Although we added sensitivity analyses and segmented trend models to characterize this pattern, the 2019 breakpoint was specified analytically and our study did not formally compare GBD 2021 and GBD 2023 model outputs; therefore, we cannot determine the extent to which the late‐period changes reflect epidemiological shifts, updated data incorporation, or changes in modeling assumptions between GBD iterations. Relatedly, our statistical analysis was fundamentally grounded in GBD 2023 estimates and therefore reflects the strengths and constraints of the GBD modeling framework. We did not generate primary epidemiological data or independently validate country‐level estimates against local surveillance systems. As a result, our findings should be interpreted as modeled estimates of disease burden rather than direct measurements of population prevalence, diagnosis, treatment access, or service utilization. This methodological reliance is particularly important in SSA, where the availability and quality of mental health research, service data, and policy documentation vary widely across countries.

Another limitation is the potential underestimation of the true burden of mental disorders in SSA, particularly due to stigma‐related underreporting. Mental health issues often carry a significant social stigma in many SSA countries, discouraging individuals from seeking help and disclosing their symptoms [47]. In addition, the poor availability of mental health services and the limited recognition of mental disorders as a public health priority by governments also contribute to these underestimates. Combined with cultural barriers, this can lead to underreporting conditions such as depression, anxiety, and substance use, resulting in a lower‐than‐actual representation of their prevalence and impact. One major factor underlying this underreporting is the low level of mental health literacy. Many individuals may not recognize symptoms of depression, anxiety, or substance use as clinical conditions, and healthcare providers, including clinicians and nurses, often lack adequate training to identify and diagnose them. Furthermore, data availability in SSA remains a critical challenge. While the GBD dataset has made significant strides in providing estimates for many countries in the region, the lack of comprehensive, high‐quality national surveys on mental health still limits our understanding of the full scope of the problem. The absence of a consistent, reliable data infrastructure in many SSA countries, particularly in rural or conflict‐affected areas, means that mental health statistics are often fragmented or incomplete, which hampers the ability to formulate effective, data‐driven health policies [44].

### 4.3. Future Research

Future research in mental health in SSA should focus on improving data collection and strengthening epidemiological surveillance systems to address the current gaps in mental health prevalence and burden estimates. There is a pressing need for national surveys and longitudinal studies to track trends over time and understand the social determinants of mental health more deeply, particularly in rural and conflict‐affected areas. In particular, future work should ensure age‐disaggregated data, allowing for more precise insights into the needs of children, adolescents, young adults, and older populations and for the development of targeted, age‐specific policies and interventions. Additionally, research should explore the impact of early life adversities such as violence and displacement on the development of mental disorders, with a focus on intergenerational trauma and its long‐term effects. Intervention studies are also needed to evaluate the effectiveness of integrating mental healthcare into existing health systems and community‐based interventions, particularly in low‐resource settings. Finally, there is a need for economic evaluations and investment case analyses to quantify the costs of inaction and highlight the potential returns of mental health interventions, thereby making the case more compelling to policymakers. This research will be critical for guiding policy development, improving service delivery, and addressing the mental health challenges facing SSA in a more targeted and sustainable manner.

## 5. Conclusion(s)

Mental disorders in SSA represent a growing public health challenge, with increasing absolute burden over the past three decades and heterogeneous age‐standardized trends across disorders. Addressing this burden will require strengthening mental health infrastructure and workforce capacity, reducing stigma, and integrating mental health services into primary and general healthcare systems. Equally important is the promotion and dissemination of preventive mental health interventions, including school‐ and community‐based mental health literacy programs, early identification of high‐risk children and adolescents, violence prevention, substance use prevention, and psychosocial support through existing community and primary care platforms. These strategies are essential to reduce future burden, improve access to care, and ensure that mental health is prioritized within health systems and policies across the region.

## Author Contributions

Basile Njei and Yazan A. Al‐Ajlouni conceptualized the research question and hypothesis. Yazan A. Al‐Ajlouni and Basile Njei conducted data analysis. Basile Njei, Yazan A. Al‐Ajlouni, Samira Y. Lemos, Nkengafac Villyen Motaze, Laurent Cleenewerck de Kiev, Luchuo E. Bain, and Yauba Saidu wrote the main manuscript text. All authors contributed to revising the work for important intellectual content. The corresponding author confirms that all listed authors meet authorship criteria and that no others meeting the criteria have been omitted.

## Funding

We received no funding for this study.

## Disclosure

All authors gave final approval of the version to be published and agreed on all aspects of the work, especially concerning its design, accuracy, and integrity.

## Ethics Statement

The authors have nothing to report.

## Conflicts of Interest

The authors declare no conflicts of interest.

## Supporting Information

Additional supporting information can be found online in the Supporting Information section.

## Supporting information


**Supporting Information** The STROBE checklist for cross‐sectional studies is provided as the supporting material. Supporting Table 1 presents the countries with the highest all‐age DALY burden attributable to mental disorders in SSA in 2023 and the countries with the largest increases in age‐standardized DALY rates from 1990 to 2023. Supporting Table 2 provides country‐level changes in all‐age DALYs and age‐standardized DALY rates attributable to mental disorders in SSA from 1990 to 2023.

## Data Availability

The data used in this study are publicly available from the IHME GHDx and GBD Results Tool. All estimates were derived from the GBD study 2023 resources.
