# Supplementary material for: Over Three Decades of Mental Health in Sub‐Saharan Africa: An Analysis of GBD Data From 1990 to 2023
Source: Glob Health Epidemiol Genom. 2026 Jul 30;2026:5570226. doi: 10.1155/ghe3/5570226 (PMC13424614; doi:10.1155/ghe3/5570226)
Supplement: Supplementary file 1 — Supporting Information The STROBE checklist for cross‐sectional studies is provided as the supporting material. Supporting Table 1 presents the countries with the highest all‐age DALY burden attributable to mental disorders in SSA in 2023 and the countries with the largest increases in age‐standardized DALY rates from 1990 to 2023. Supporting Table 2 provides country‐level changes in all‐age DALYs and age‐standardized DALY rates attributable to mental disorders in SSA from 1990 to 2023. [file GHE3-2026-5570226-s001.docx]

**Supplementary File 1**

STROBE Statement—Checklist of items that should be included in reports of ***cross-sectional studies***

|  | Item No | Recommendation | Page No |
| --- | --- | --- | --- |
| **Title and abstract** | 1 | (*a*) Indicate the study’s design with a commonly used term in the title or the abstract | 1 |
|  |  | (*b*) Provide in the abstract an informative and balanced summary of what was done and what was found | 2 |
| Introduction | | | |
| Background/rationale | 2 | Explain the scientific background and rationale for the investigation being reported | 4, 5 |
| Objectives | 3 | State specific objectives, including any prespecified hypotheses | 5 |
| Methods | | | |
| Study design | 4 | Present key elements of study design early in the paper | 5, 6 |
| Setting | 5 | Describe the setting, locations, and relevant dates, including periods of recruitment, exposure, follow-up, and data collection | 5, 6 |
| Participants | 6 | (*a*) Give the eligibility criteria, and the sources and methods of selection of participants | 5, 6 |
| Variables | 7 | Clearly define all outcomes, exposures, predictors, potential confounders, and effect modifiers. Give diagnostic criteria, if applicable | 5, 6 |
| Data sources/ measurement | 8* | For each variable of interest, give sources of data and details of methods of assessment (measurement). Describe comparability of assessment methods if there is more than one group | 5, 6 |
| Bias | 9 | Describe any efforts to address potential sources of bias | 5, 6 |
| Study size | 10 | Explain how the study size was arrived at | 6 |
| Quantitative variables | 11 | Explain how quantitative variables were handled in the analyses. If applicable, describe which groupings were chosen and why | 6 |
| Statistical methods | 12 | (*a*) Describe all statistical methods, including those used to control for confounding | 6 |
|  |  | (*b*) Describe any methods used to examine subgroups and interactions | 6 |
|  |  | (*c*) Explain how missing data were addressed | 5 |
|  |  | (*d*) If applicable, describe analytical methods taking account of sampling strategy | N/A |
|  |  | (*e*) Describe any sensitivity analyses | N/A |
| Results | | | |
| Participants | 13* | (a) Report numbers of individuals at each stage of study—eg numbers potentially eligible, examined for eligibility, confirmed eligible, included in the study, completing follow-up, and analysed | N/A |
|  |  | (b) Give reasons for non-participation at each stage | N/A |
|  |  | (c) Consider use of a flow diagram | N/A |
| Descriptive data | 14* | (a) Give characteristics of study participants (eg demographic, clinical, social) and information on exposures and potential confounders | 7-9 |
|  |  | (b) Indicate number of participants with missing data for each variable of interest | N/A |
| Outcome data | 15* | Report numbers of outcome events or summary measures | 7-9 |
| Main results | 16 | (*a*) Give unadjusted estimates and, if applicable, confounder-adjusted estimates and their precision (eg, 95% confidence interval). Make clear which confounders were adjusted for and why they were included | 7-9 |
|  |  | (*b*) Report category boundaries when continuous variables were categorized | 9 |
|  |  | (*c*) If relevant, consider translating estimates of relative risk into absolute risk for a meaningful time period | N/A |
| Other analyses | 17 | Report other analyses done—eg analyses of subgroups and interactions, and sensitivity analyses | N/A |
| Discussion | | | |
| Key results | 18 | Summarise key results with reference to study objectives | 9, 10 |
| Limitations | 19 | Discuss limitations of the study, taking into account sources of potential bias or imprecision. Discuss both direction and magnitude of any potential bias | 14, 15 |
| Interpretation | 20 | Give a cautious overall interpretation of results considering objectives, limitations, multiplicity of analyses, results from similar studies, and other relevant evidence | 10-15 |
| Generalisability | 21 | Discuss the generalisability (external validity) of the study results | 14, 15 |
| Other information | | | |
| Funding | 22 | Give the source of funding and the role of the funders for the present study and, if applicable, for the original study on which the present article is based | 17 |

**Supplementary Table 1.** Countries with the highest all-age DALYs attributable to mental disorders in Sub-Saharan Africa in 2023.

| Rank | Ranking basis | Country | All-age DALYs 1990 | All-age DALYs 2023 | % change in all-age DALYs | ASDR 1990 | ASDR 2023 | % change in ASDR |
| --- | --- | --- | --- | --- | --- | --- | --- | --- |
| 1 | Largest 2023 all-age DALY burden | Nigeria | 1,233,749 | 5,377,571 | 335.87% | 1,532.2 | 2,289.3 | 49.41% |
| 2 | Largest 2023 all-age DALY burden | Ethiopia | 762,734 | 2,365,758 | 210.17% | 1,796.0 | 2,246.0 | 25.06% |
| 3 | Largest 2023 all-age DALY burden | Democratic Republic of the Congo | 608,114 | 2,071,443 | 240.63% | 1,856.8 | 2,426.0 | 30.66% |
| 4 | Largest 2023 all-age DALY burden | South Africa | 580,239 | 1,404,326 | 142.03% | 1,636.9 | 2,117.6 | 29.37% |
| 5 | Largest 2023 all-age DALY burden | United Republic of Tanzania | 323,794 | 1,138,203 | 251.52% | 1,452.9 | 1,915.3 | 31.83% |
| 6 | Largest 2023 all-age DALY burden | Kenya | 300,033 | 952,436 | 217.44% | 1,513.4 | 1,883.0 | 24.42% |
| 7 | Largest 2023 all-age DALY burden | Angola | 193,418 | 860,312 | 344.79% | 2,143.3 | 2,663.4 | 24.27% |
| 8 | Largest 2023 all-age DALY burden | Uganda | 230,745 | 854,815 | 270.46% | 1,586.9 | 2,062.4 | 29.96% |
| 9 | Largest 2023 all-age DALY burden | Cameroon | 149,807 | 736,110 | 391.37% | 1,639.0 | 2,294.9 | 40.01% |
| 10 | Largest 2023 all-age DALY burden | Côte d'Ivoire | 169,134 | 734,259 | 334.13% | 1,571.8 | 2,245.9 | 42.88% |
| 1 | Largest 1990–2023 ASDR increase | Nigeria | 1,233,749 | 5,377,571 | 335.87% | 1,532.2 | 2,289.3 | 49.41% |
| 2 | Largest 1990–2023 ASDR increase | Mali | 112,777 | 492,756 | 336.93% | 1,511.4 | 2,245.5 | 48.57% |
| 3 | Largest 1990–2023 ASDR increase | Niger | 99,773 | 514,175 | 415.35% | 1,487.8 | 2,198.1 | 47.74% |
| 4 | Largest 1990–2023 ASDR increase | Gambia | 13,417 | 55,494 | 313.61% | 1,561.4 | 2,280.5 | 46.06% |
| 5 | Largest 1990–2023 ASDR increase | Guinea | 85,717 | 313,794 | 266.08% | 1,634.9 | 2,376.9 | 45.39% |
| 6 | Largest 1990–2023 ASDR increase | Guinea-Bissau | 13,538 | 45,666 | 237.32% | 1,538.5 | 2,221.6 | 44.40% |
| 7 | Largest 1990–2023 ASDR increase | Burkina Faso | 136,192 | 527,132 | 287.05% | 1,668.0 | 2,400.1 | 43.89% |
| 8 | Largest 1990–2023 ASDR increase | Chad | 91,732 | 436,368 | 375.70% | 1,803.8 | 2,589.7 | 43.57% |
| 9 | Largest 1990–2023 ASDR increase | Côte d'Ivoire | 169,134 | 734,259 | 334.13% | 1,571.8 | 2,245.9 | 42.88% |
| 10 | Largest 1990–2023 ASDR increase | Cabo Verde | 4,964 | 11,551 | 132.67% | 1,587.5 | 2,261.9 | 42.48% |

**Supplementary Table 2.** Country-level changes in all-age DALYs and age-standardized DALY rates for mental disorders in Sub-Saharan Africa, 1990–2023.

| Country | All-age DALYs 1990 | All-age DALYs 2023 | % change in all-age DALYs | ASDR 1990 | ASDR 2023 | % change in ASDR |
| --- | --- | --- | --- | --- | --- | --- |
| Angola | 193,418 | 860,312 | 344.79% | 2,143.3 | 2,663.4 | 24.27% |
| Benin | 70,124 | 305,325 | 335.41% | 1,693.6 | 2,307.0 | 36.22% |
| Botswana | 18,666 | 48,306 | 158.79% | 1,580.4 | 1,981.8 | 25.40% |
| Burkina Faso | 136,192 | 527,132 | 287.05% | 1,668.0 | 2,400.1 | 43.89% |
| Burundi | 75,232 | 260,195 | 245.86% | 1,587.5 | 2,025.7 | 27.60% |
| Cabo Verde | 4,964 | 11,551 | 132.67% | 1,587.5 | 2,261.9 | 42.48% |
| Cameroon | 149,807 | 736,110 | 391.37% | 1,639.0 | 2,294.9 | 40.01% |
| Central African Republic | 45,281 | 153,851 | 239.77% | 1,884.6 | 2,665.6 | 41.44% |
| Chad | 91,732 | 436,368 | 375.70% | 1,803.8 | 2,589.7 | 43.57% |
| Comoros | 6,510 | 18,730 | 187.71% | 1,600.6 | 2,200.4 | 37.47% |
| Congo | 41,406 | 148,237 | 258.01% | 1,931.1 | 2,550.3 | 32.06% |
| Côte d'Ivoire | 169,134 | 734,259 | 334.13% | 1,571.8 | 2,245.9 | 42.88% |
| Democratic Republic of the Congo | 608,114 | 2,071,443 | 240.63% | 1,856.8 | 2,426.0 | 30.66% |
| Djibouti | 5,365 | 26,985 | 403.01% | 1,512.0 | 2,000.3 | 32.30% |
| Equatorial Guinea | 6,706 | 37,726 | 462.60% | 1,854.3 | 2,431.2 | 31.11% |
| Eritrea | 47,684 | 131,618 | 176.02% | 1,626.3 | 1,993.8 | 22.60% |
| Eswatini | 11,493 | 24,840 | 116.12% | 1,634.6 | 2,123.5 | 29.91% |
| Ethiopia | 762,734 | 2,365,758 | 210.17% | 1,796.0 | 2,246.0 | 25.06% |
| Gabon | 16,995 | 46,713 | 174.86% | 1,913.5 | 2,538.7 | 32.68% |
| Gambia | 13,417 | 55,494 | 313.61% | 1,561.4 | 2,280.5 | 46.06% |
| Ghana | 216,113 | 722,254 | 234.20% | 1,629.7 | 2,240.6 | 37.48% |
| Guinea | 85,717 | 313,794 | 266.08% | 1,634.9 | 2,376.9 | 45.39% |
| Guinea-Bissau | 13,538 | 45,666 | 237.32% | 1,538.5 | 2,221.6 | 44.40% |
| Kenya | 300,033 | 952,436 | 217.44% | 1,513.4 | 1,883.0 | 24.42% |
| Lesotho | 19,850 | 38,435 | 93.62% | 1,455.6 | 2,013.0 | 38.30% |
| Liberia | 39,029 | 133,818 | 242.87% | 1,669.7 | 2,371.3 | 42.02% |
| Madagascar | 156,869 | 583,484 | 271.96% | 1,515.3 | 2,052.1 | 35.42% |
| Malawi | 126,256 | 398,948 | 215.98% | 1,496.5 | 2,048.8 | 36.91% |
| Mali | 112,777 | 492,756 | 336.93% | 1,511.4 | 2,245.5 | 48.57% |
| Mauritania | 26,656 | 89,359 | 235.24% | 1,467.4 | 2,036.4 | 38.77% |
| Mozambique | 208,561 | 643,795 | 208.68% | 1,773.6 | 2,160.6 | 21.82% |
| Namibia | 18,388 | 56,593 | 207.76% | 1,440.4 | 1,919.4 | 33.26% |
| Niger | 99,773 | 514,175 | 415.35% | 1,487.8 | 2,198.1 | 47.74% |
| Nigeria | 1,233,749 | 5,377,571 | 335.87% | 1,532.2 | 2,289.3 | 49.41% |
| Rwanda | 93,364 | 274,822 | 194.36% | 1,507.5 | 2,060.6 | 36.69% |
| Sao Tome and Principe | 1,706 | 5,089 | 198.24% | 1,586.0 | 2,254.8 | 42.17% |
| Senegal | 102,781 | 386,380 | 275.93% | 1,556.2 | 2,154.7 | 38.46% |
| Sierra Leone | 58,870 | 178,298 | 202.87% | 1,635.7 | 2,292.7 | 40.16% |
| Somalia | 108,830 | 366,275 | 236.56% | 1,595.9 | 1,949.2 | 22.14% |
| South Africa | 580,239 | 1,404,326 | 142.03% | 1,636.9 | 2,117.6 | 29.37% |
| South Sudan | 83,428 | 205,445 | 146.25% | 1,624.5 | 2,221.5 | 36.75% |
| Togo | 60,960 | 223,907 | 267.30% | 1,888.4 | 2,677.4 | 41.78% |
| Uganda | 230,745 | 854,815 | 270.46% | 1,586.9 | 2,062.4 | 29.96% |
| United Republic of Tanzania | 323,794 | 1,138,203 | 251.52% | 1,452.9 | 1,915.3 | 31.83% |
| Zambia | 106,544 | 411,817 | 286.52% | 1,558.1 | 2,140.1 | 37.36% |
| Zimbabwe | 148,931 | 300,107 | 101.51% | 1,630.6 | 1,993.0 | 22.23% |
